# Network pharmacology reveals *Ficus. Carica. L* latex as a potential therapeutic agent for gastric ulcers by modulating inflammation and promoting repair

**DOI:** 10.1371/journal.pone.0333777

**Published:** 2025-12-02

**Authors:** Nourhan Hisham Shady, Mohamed Hisham, Hend Samy Abdullah, Sherif A. Maher, Amgad Albohy, Mahmoud A. Elrehany, Ahmed M. Sayed, Esam S. Allehyani, Manal M. Khowdiary, Ahmed M. Shawky, Usama Ramadan Abdelmohsen

**Affiliations:** 1 Department of Pharmacognosy, Faculty of Pharmacy, Deraya University, Universities Zone, New Minia City,; 2 Center for Research and Sustainability, Deraya University, Universities Zone, New Minia City, Egypt; 3 Department of Pharmaceutical Chemistry, Faculty of Pharmacy, Deraya University, New-Minia, Egypt; 4 Faculty of Pharmacy, Deraya University, Universities Zone, New Minia City, Egypt; 5 Department of Biochemistry, Faculty of Pharmacy, New valley University, Universities Zone, New Valley, EL-kharga City,; 6 Department of Medicinal Chemistry, College of Pharmacy, University of Sharjah, Sharjah, United Arab Emirates; 7 Department of Pharmaceutical Chemistry, Faculty of Pharmacy, The British University in Egypt, El-Sherouk City, Cairo,; 8 The Center for Drug Research and Development (CDRD), Faculty of Pharmacy, The British University in Egypt, El-Sherouk City, Cairo, Egypt; 9 Department of Biochemistry, Faculty of Pharmacy, Deraya University, Universities Zone, New Minia City, Egypt; 10 Department of Pharmacognosy, Faculty of Pharmacy, Almaaqal University, 61014 Basra, Iraq; 11 Department of Chemistry, University College in Al-Jamoum, Umm Al-Qura University, Makkah, Saudi Arabia; 12 Department of Chemistry, Faculty of Applied Science, Lieth Collage, Umm Al-Qura University, Makkah, Saudi Arabia; 13 Department of Chemistry, Faculty of Science, Umm Al-Qura University, Makkah, Saudi Arabia; 14 Department of Pharmacognosy, Faculty of Pharmacy, Minia University, Minia, Egypt; Cairo University, Faculty of Science, EGYPT

## Abstract

The primary objective of our research is to investigate the gastroprotective impact of *Ficus. carica L.* latex extract (FCL). Metabolic profiling based on HR- LCMS for the extract led to the annotation of 20 compounds. Additionally, the gastro-protective activity of the latex extract was evaluated in vivo using male albino rats. FCL significantly alleviated the indomethacin-induced ulceration and reduced the ulcer index. Furthermore, the inflammation caused by indomethacin was seen to diminish due to the downregulation of the production of certain genes (*IL-6, and TNF-α, IL-1β*). Besides, FCL considerably reduced the elevated *TGF-β, and IGF-1, COX-2* relative gene expression. Likewise, FCL dramatically raised *EGF* and *KGF* relative gene expression, demonstrating its beneficial impact in ulcer healing. The antioxidant ability of FCL was assessed by in vitro experiments utilizing hydrogen peroxide and superoxide radical scavenging, which demonstrated significant antioxidant capacity. Employing network pharmacology, we identified 10 hub genes central to peptic ulcer disease and conducted molecular docking studies screening interactions between FCL extract compounds and these hubs along with anti-inflammatory targets including FGFR, TGF-βR, IGFR-1R and IL-1R1 involved in ulcer healing. Additionally, an in-silico study using annotated FCL compounds highlighted Ficuseptin-C, Ficuseptin-B and Ficusin-A in the extract may contribute to its antiulcer properties. The present study highlighted the potential of FCL as a promising natural gastro-protective agent.

## 1. Introduction

Gastric ulcer is a frequent digestive system condition [1]. Peptic ulcers are persistent, localized lesions that develop in any segment of the gastrointestinal system subjected to the aggressive effects of acid-peptic secretions [2]. A peptic ulcer develops when there is a disruption in the balance between internal factors that promote damage—such as hydrochloric acid, pepsin, bile reflux, leukotrienes, reactive oxygen species (ROS)—and protective mechanisms, which include the mucus-bicarbonate layer, surface phospholipids, prostaglandins (PGs), mucosal blood circulation, cell regeneration and migration, along with both enzymatic and non-enzymatic defense systems. [3].The two major variables affecting mucosal tolerance to damage are non-steroidal anti-inflammatory medications (NSAIDs) and *Helicobacter pylori* (*H. pylori*) infection. Pharmacological therapies such as proton pump inhibitors, H2-blockers, anti *H.pylori* drugs, antacids, etc. [4]. There are a variety of medicines available to treat peptic ulcers, but clinical trials show that they have a high risk of relapse, as well as adverse effects and drug interactions [5].So, in terms of PU management/treatment, herbal medicine is increasingly becoming a viable alternative to commercially available synthetic medicines [6]. Stimulation of mucous cell proliferation, anti-oxidation, and suppression of gastric acid production, as well as H (+)/K (+)-ATPase activity, are some of the processes by which herbal medications improve gastric ulcers [1]. Several medicinal herbs have been shown to have strong anti-ulcer properties [2]. Natural products play avital role as gastroprotective agents [2,7,8]. *Ficus* is a genus containing around 850 species in the *Moraceae* plant family. The majority of *Ficus* species are consumed by humans as a source of nourishment [9]. Because of The presence of diverse bioactive phytochemical compounds, including phenolic acids, polyphenols, flavonoids, triterpenoids, flavonols, anthocyanins, carotenoids, glycosides, polysaccharides, reducing compounds, and vitamins K, E, and C, in various plant parts such as fruits, seeds, leaves, tender shoots, bark, and latex, has numerous medicinal applications [9,10]. It has been used for several ailments affecting the digestive, endocrine, reproductive, and respiratory systems in traditional medicine. It is further used to address gastrointestinal and urinary tract infections [11]. *F. carica* is capable of a wide range of biological functions. *F. carica* was used in traditional medicine to treat ulcers, indigestion, and diarrhea, according to reports. A wide range of pharmacological properties, including antibacterial, antioxidant, anticancer, and anti-inflammatory effects, have been reported in several published research studies [12]. The common *Ficus. carica.L* Latex., is widely used in food and medicine in the Middle East [13]. Latex is a material that forms when immature fig tree leaves are broken, and it contains the cysteine proteinase enzyme, which is active in the pH range of 6.5–8.5.9 [14]. Cancer, inflammatory disorders, bacterial and gastrointestinal nematode infections, warts, gout, ulcers, and skin problems are all treated using fig latex [15,16]. Hence, our study was designed to assess the efficacy of *Ficus. carica L* L against indomethacin-induced peptic ulcers in rats.

## 2. Result and discussion

### 2.1. Antioxidant activity of FCL *in Vitro*

#### 2.1.1. Activity of hydrogen peroxide scavenging.

**Fig 1:**
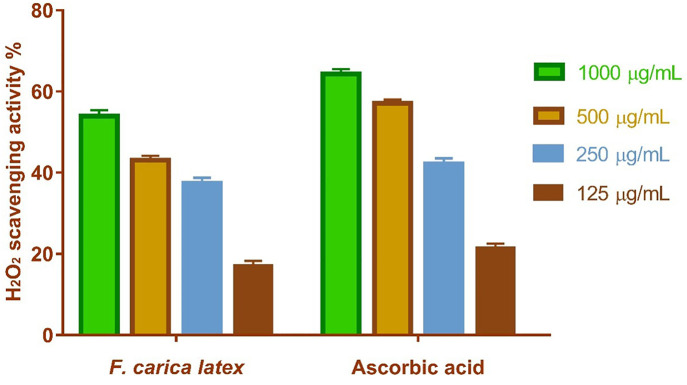
*Ficus Carica* extract H_2_O_2_ radical scavenging activity at various concentrations (1000 ug/ml, 500 ug/ml, 250 ug/ml, 125 ug/ml). The bars reflect the mean and standard deviation (SD). A Two-way ANOVA test is used to determine whether there is a significant difference between groups.

This research examined the antioxidant efficacy of FCL as a potential scavenger of H_2_O_2_. The maximum H_2_O_2_ radical scavenging activity of FCL was 53.93% at a concentration of 1000 µg/mL, as per the data. FCL decreased H_2_O_2_ generation in a dose-dependent manner (IC50 = 179.6 µg/mL) relative to normal ascorbic acid (IC50 = 181 µg/mL) which confirms the strong activity of hydrogen peroxide scavenging of FCL with IC_50_ very close to IC_50_ of ascorbic acid which is used as internal standard control (Fig 1).

#### 2.1.2. Activity of superoxide radical scavenging.

**Fig 2:**
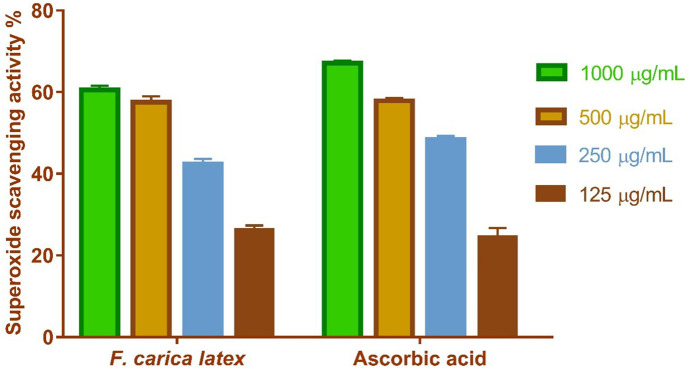
FCL superoxide radicle scavenging activity at various concentrations (1000 ug/ml, 500 ug/ml, 250 ug/ml, 125 ug/ml). The bars reflect the mean and standard deviation (SD). A Two-way ANOVA test is used to determine whether there is a significant difference between groups.

FCL was evaluated for superoxide dismutase (SOD) activity. The superoxide radical scavenging activity of FCL was concentration-dependent, with the maximum efficacy seen at a concentration of 1000 μg/mL, when FCL exhibited a 60.6% superoxide scavenging efficiency. The IC_50_ concentration of FCL for 50% inhibition was established at 149.6 μg/mL, in comparable to ascorbic acid, which had an IC_50_ of 159.5 μg/mL as shown in Fig 2.

### 2.2. *FCL*’s impact on IL-1β, TNF-α, TGF-β, IL-6, EGF, IGF-1, COX-2, and KGF expression

The healing of a stomach ulcer after mucosal damage involves a complex physiological process that encompasses tissue auto-repair, reperfusion, and regeneration. A digestive tract ulcer is a lesion composed of granular and connective tissues, macrophages, and fibroblasts, accompanied by angiogenesis, leading to the development of proliferating microvessels at the ulcer’s base and margins [17]. The non-necrotic mucosa around this lesion forms an ulcerative margin that heals excessively rapidly, resulting in an ulcer scar. The process of ulcer development consists of four phases: Indomethacin-induced inflammation, tissue necrosis, infiltration of inflammatory cells, ulcer formation, and ultimately, the development of granulation tissue and ulcer margins [18].

**Fig 3:**
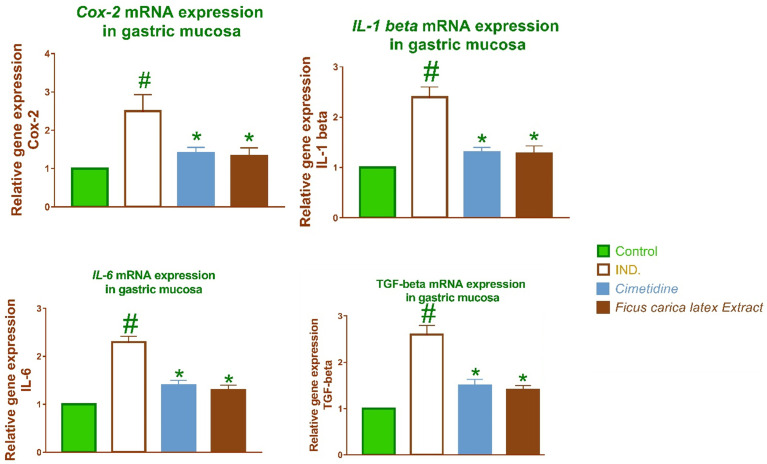
The relative gene expression of COX-2, IL-1β, TGF- β in gastric mucosa tissue of different groups of rats was determined using quantitative RT-PCR. The findings after normalization to GAPDH show a fold difference in expression relative to the normal control group. The bars reflect the mean and standard deviation (SD). A Two-way ANOVA test is used to determine whether there is a significant difference between groups with # p < 0.05 compared to the normal control group and * p < 0.05 compared to the indomethacin induced group.

**Fig 4:**
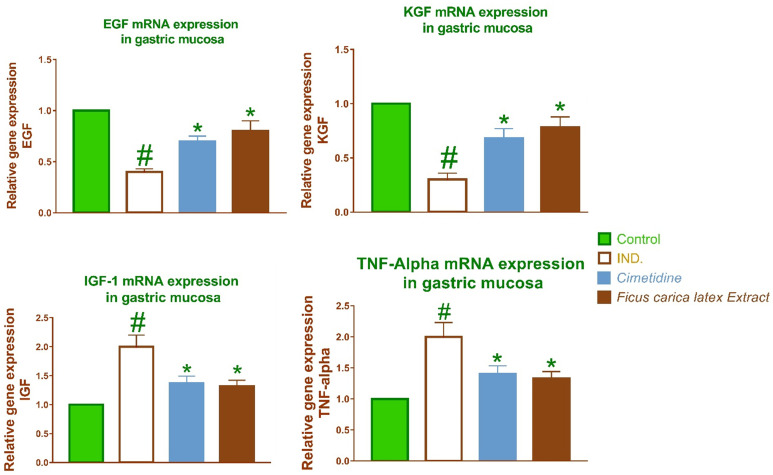
Using quantitative RTPCR, the relative gene expression of EGF, KGF, IGF-1 and TNFα in the gastric mucosa tissue of several groups of rats was evaluated. The findings show a fold difference in expression when compared to the normal control group after being normalized to GAPDH. The bars display the standard deviation from the mean. A One-way ANOVA test is used to determine whether there is a significant difference between groups, with # p < 0.05 compared to the normal control group and * p < 0.05 compared to the indomethacin induced group.

Macrophages secrete tumor necrosis factor-alpha (TNF-α), interleukin-6 (IL-6), and interleukin-1 beta (IL-1β), upon activation of immune cells. The proinflammatory cytokine *IL-1β* modulates several genes associated with tissue damage and inflammation, including those influencing the functionality of entero-chromaffin-like cells. Moreover, protein kinase C, tyrosine kinase, in addition to several other mitogen-activated protein kinases affect the decrease in gastric epithelial cell proliferation induced by *IL-1β*. Fig 3 and Fig 4. illustrate the antiulcer action of *Ficus. Carica. L* latex extract may mitigate the inflammation induced by TNF-α, *IL-6*, and *IL-1β* by reducing subsequent inflammatory responses.

Indomethacin-induced gastritis correlates with *COX-2* activation in epithelial, fibroblasts cells, mononuclear cells, and parietal cells of the mucosa, as well as in reaction to elevated *IL-1βeta* levels. The findings indicated that *Ficus. Carica.L* latex extract markedly decreased the elevated relative *COX-2* expression, hence affirming its preventive role in ulcer to be healed, as seen in Fig 3 and Fig 4. *TNF-α* by attenuating subsequent inflammatory reactions.

In indomethacin-induced ulcers in the rat stomach, *IL-1β* facilitates the early elevation of *TGF-β* and *IGF-1* [19]. *TGF-βeta* inhibits cell proliferation in certain cells, however, it also facilitates cell migration, angiogenesis, in addition to ECM (Extracellular Matrix) formation, all of which are crucial for gastrointestinal ulcer healing during the intermediate and late stages. It has anti-inflammatory effects [19]. After indomethacin-induced inflammation, macrophages are activated, enhancing the release of active *TGF-β* and the features of plasmin activation [20].

Moreover, IGF-1 expression has been associated with the expedited healing of ulcers in both the initial and later phases. IGF-1, a crucial regulator of soft tissue regeneration, is rapidly synthesized from platelet granules upon damage and is vital in gastric ulcer angiogenesis and epithelialization [21]. Consequently, we found that the IGF-1 mRNA level was markedly increased in rats with Indomethacin-induced stomach injury, as shown in Fig 4. Ultimately, treatment with *Ficus. carica L* latex extract resulted in a notable reduction of the elevated levels of relative *IGF-1*and *TGF-β* gene expression levels.

The *KGF* gene has a greater protection impact on the gastrointestinal tract of ulcerative colitis in rats, perhaps due to its reparative properties [22]. *KGF* belongs to the Fibroblast Growth Factors (FGFs) family, which facilitates the repairing of tissue damage. It is regarded as a modulator of mesenchymal-epithelial interactions, that may increase epithelial cell differentiation and proliferation by affecting the interaction between MSCs and epithelial cells [23]. *KGF* significantly decreased damage to intestinal epithelial crypts and improved intestinal mucosal tolerance. Consequently, we reported that the *KGF* gene level was markedly reduced in the gut mucosa of normal rats after ulceration. Ultimately, after treatment with *Ficus. carica L* latex extract, the results demonstrated a significant elevation in the gene expression of high *KGF,* as seen in Fig 4. *EGF* has the capacity to stimulate epithelial proliferation, facilitate tissue healing, in addition to give cell protection. It is crucial for safeguarding the gastric mucosa from harmful agents and maintaining the integrity of the gastrointestinal mucosa [24]. Our data indicates that the relative gene expression of *EGF* in the stomach tissue of rats with Indomethacin-induced gastric injury was much lower than that of normal rats. *EGF* has been shown to enhance ulcer healing, augment tissue repair capacity, and maintain the quality of ulcer healing as a protective factor [25]. The present study demonstrates that the administration of *Ficus. Carica. L* latex extract markedly elevated the expression of the *EGF* gene in the gastric tissue of rats with gastric injury, suggesting a protective effect of the extract on the gastric tissue, as illustrated in Fig 4.The biological significance of upregulating *EGF* and *KGF* and downregulating *TGF-β* and *IGF-1* lies in promoting mucosal healing, limiting fibrosis, and restoring the gastric epithelial barrier. Each of these factors plays a distinct role in the repair and regeneration of the gastric mucosa. Upregulating *EGF* in Gastric Ulcers accelerates re-epithelialization of the ulcer, promotes cell survival and tissue regeneration, and reduces the healing time and recurrence risk. Upregulating *KGF* in Gastric Ulcers supports the regeneration of gastric mucosa and glandular structures, reinforces the gastric barrier against acid and pepsin, and improves ulcer healing quality by restoring normal epithelial architecture. Downregulating *TGF-β* in Gastric Ulcers helps prevent fibrotic healing and strictures, promotes balanced regeneration over excessive matrix formation, and reduces risk of dysplasia linked to chronic *TGF-β* activation. And Downregulating *IGF-1* in Gastric Ulcers reduces the risk of hyperplasia or metaplasia during chronic ulcer repair and helps ensure regulated healing without promoting precancerous changes in gastric mucosa. This regulation strategy enhances gastric ulcer healing by Promoting proliferation and migration of gastric epithelial cells (EGF, KGF), reducing fibrosis and scarring (TGF-β), and preventing hyperplasia and tumor risk (IGF-1).

### 2.3. Histopathological examination

**Fig 5:**
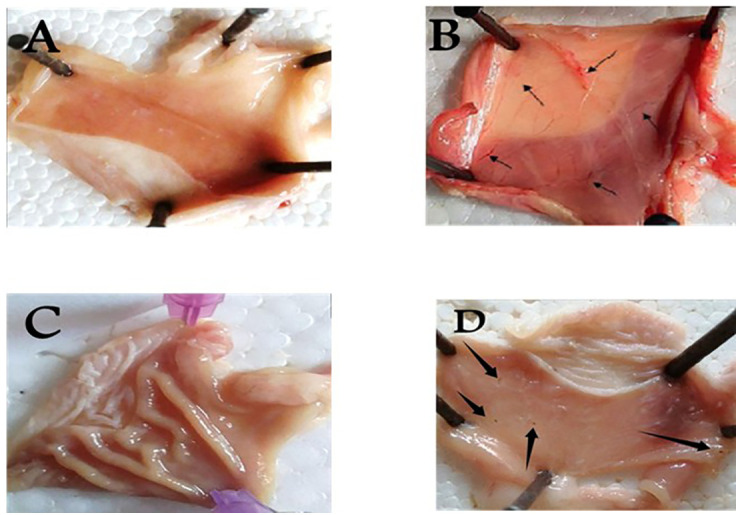
(A–D) Photos of rat stomachs. (A) (normal group), (B) (positive control group), (C) (cimetidine group), (D) (*Ficus Carica* extract group). Effect of *Ficus Carica* extract on the gastric lesion severity (gross examination). (A) Control: intact gastric mucosa tissues; (B) indomethacin (ulcer): severe lesions are seen with extensive visible hemorrhagic necrosis of gastric mucosa; (C) cimetidine-treated rats: mild lesions of the gastric mucosa are observed compared to the lesions in indomethacin-induced ulcer rats; (D) *Ficus Carica* extract -treated rats: nearly normal gastric mucosa tissues.

**Fig 6:**
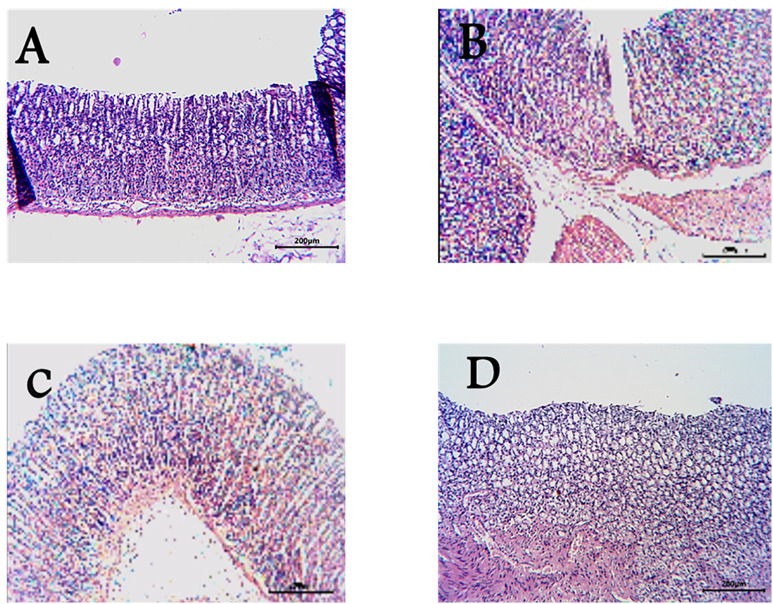
Light micrographs showing the effect of *Ficus Carica* extract on Indomethacin-induced gastric ulcers of rats. **(A)** Group A (normal mucosa), **(B)** Group B (Indomethacin-induced gastric mucosal lesions), **(C)** Group C (cimetidine treated group show low mucosal lesions), **(D)** Group D (pretreatment of rats with *Ficus Carica* extract) show no mucosal alterations.

Histopathological investigation of the removed stomachs indicated a similar pattern of ulcer severity based on the gross examinations. Where all photos of the rat stomach were explained in Fig 5. And the histopathological investigations were explained in Fig 6.

### 2.4. Metabolic profiling

**Fig 7:**
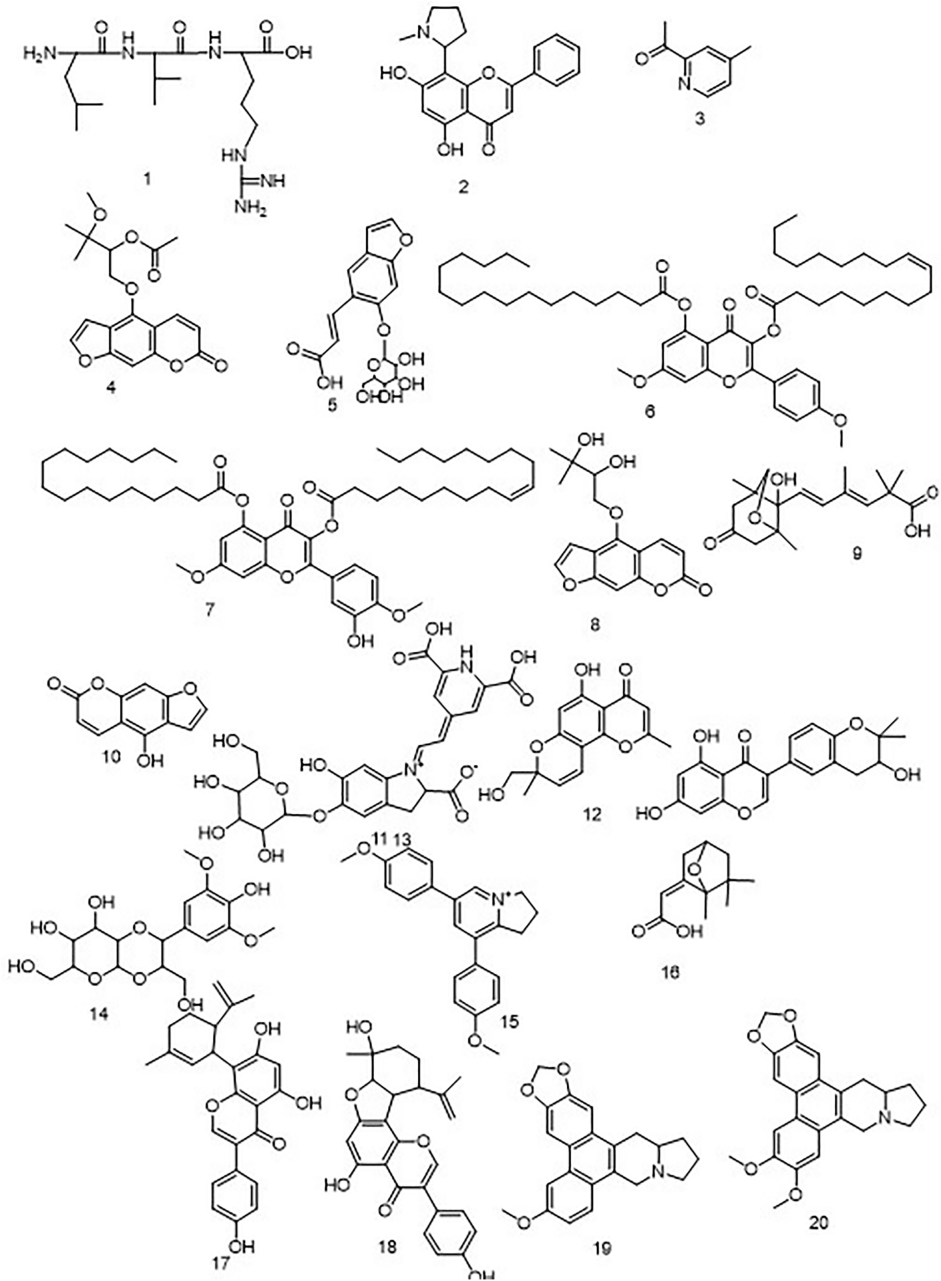
Putative compounds identified from *Ficus. carica L latex.*

Metabolic profiling by HR-LCMS was conducted on the crude extract of *Ficus. carica L* latex to examine the chemical contents potentially responsible for its preventive effects against ulcer development. The chemicals discovered in Fig 7. were categorized into several chemical classes. The identification of the chemicals was accomplished by HR-ESIMS data and comparison with existing literature. Ficus Latex peptide **3 (1)** [26], Ficine (**2**) [27], 2-Acetyl-4-methylpyridine (**3**) [28], Aviprin E- form 3’-Me ether, 2’-Ac (**4**) [29], Psoralenoside (**5**) [30], Caricaflavonol diester A (**6**) [31], Caricaflavonol diester B (**7**) [31], Aviprin (**8**) [29], Benganoic acid (**9**) [32], Bergaptol (**10**) [33], Neobetanin (**11**) [34], Ficuformodiol A (**12**) [35], Ficuisoflavone (**13**) [36], Ficuscarpanoside B (**14**) [37], Ficuseptine (**15)** [38], Ficusic acid (**16**) [39], Ficusin A (**17**) [40], Ficusin B (**18**) [40], Ficuseptine C (**19**) [41], Ficuseptine B (**20**) [41] as shown in Table S1 Fig in S1 File 7. *Ficus. Carica. L* latex offered significant dose-dependent protection against indomethacin’s ulcerogenic effects (Table 1). We conducted the research in the following way: all of the animals treated with absolute indomethacin at a dose of (5 mg/kg) developed ulcers. In the different treatment groups, gross inspection of the removed rat stomachs revealed varied degrees of ulceration. Group A was a typical control group, which has not any ulcers, while Group B (IND) had the most severe ulcers, and group C (cimetidine) clearly showed mild lesions. Groups D (MFCL) had the fewest ulcers Fig 5. The ulcer index of the animals in the negative control group (IND) was the greatest (117.32 ± 23.4), while the ulcer index of group C (2.0 ± 1.01) and group D was the lowest (1.333 ± 2.309). (Table 1). Similarly, the group given methanolic extract of *Ficus. Carica. L* latex showed a greater ulcer preventative index (96.59%).

**Table 1:** Antiulcer activity of *Ficus. carica L* latex.

Group	Level 1	Level 2	Level 3	Ulcer index	PI %
Normal	–	–	–	–	–
Indomethacin	22 ± 3.04	26.67 ± 2.17	14 ± 5.28	117.32 ± 23.4	–
IND+ cimetidine	1.31 ± 0.32	0.3 ± 0.31	0	2.0 ± 1.01	98.2%
IND + MFCL	2 ± 1.73	0	0	1.33 ± 2.3	96.59%

### 2.5. Network pharmacology-based analysis of *Ficus. carica L* latex extract in peptic ulcer

Building a gene network focused on peptic ulcer disease is important to better understand the genetic factors involved in the condition and develop personalized treatments. A well-designed gene network can reveal key molecular mechanisms of peptic ulcer disease and uncover potential new drug targets. After constructing the network, docking studies could examine the most relevant peptic ulcer targets and how they might interact with phytochemical compounds.

#### 2.5.1. Screening of *F.carica* latex extract related targets genes.

A total of 466 target genes associated with compounds found in the latex extract of *Ficus. carica L* were retrieved from the BATMAN-TCM database and Swiss Target Prediction. These genes were then standardized to their official gene names using the UniProt database.

#### 2.5.2. Screening of peptic ulcer related genes.

**Fig 8:**
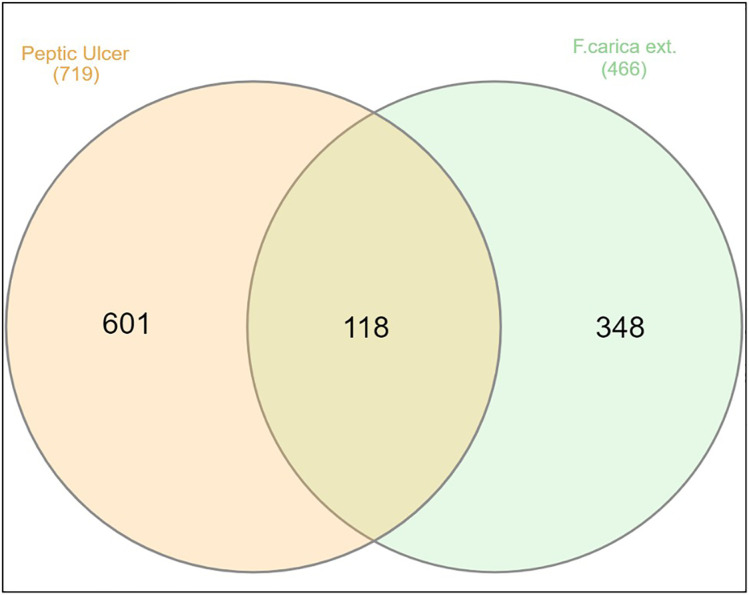
Venn diagram for the integrated.

719 target genes known to be associated with peptic ulcer disease were collected from the GeneCards, CTD, and Disgenet databases using the search terms “**peptic ulcer**” and limiting results to Homo sapiens. Duplicate targets were removed. A Venn diagram was created to compare the targets regulated by compounds extracted from *Ficus. Carica. L* latex and the potential targets for peptic ulcer disease. This revealed a total of 118 genes that were common between the two target sets Fig 8.

**Fig 9:**
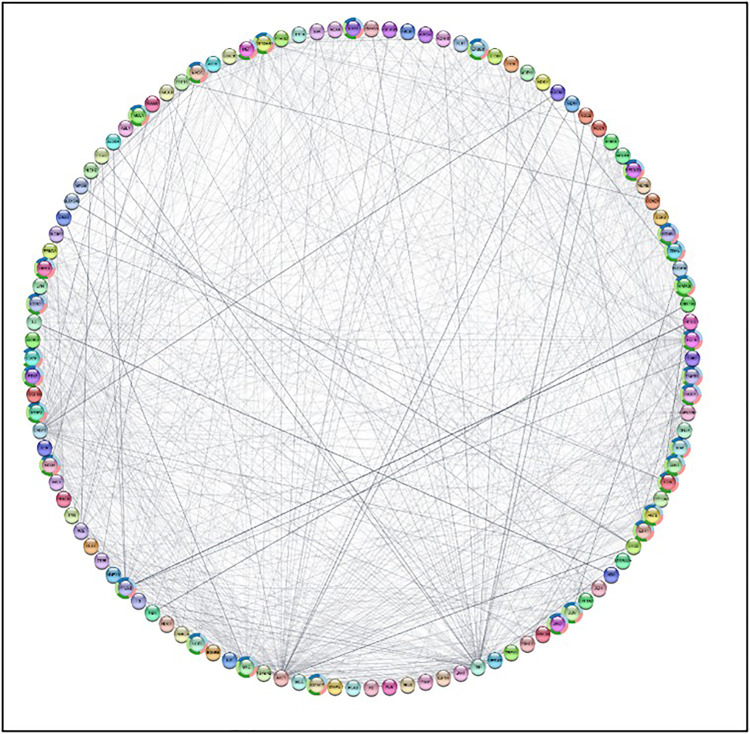
Network nodes represent 117 protein targets, and the edges represent protein-protein interactions. The size of nodes signifies the connectivity of each protein, the higher the node size the higher its connectivity to other nodes.

**Fig 10:**
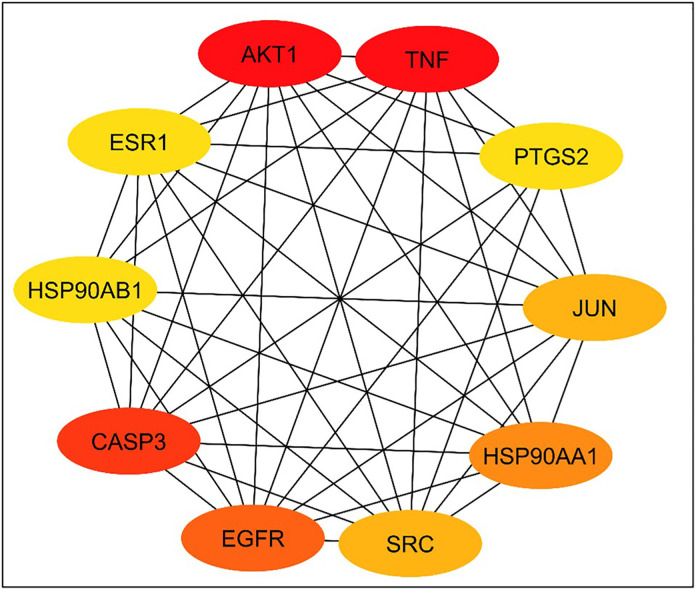
Network nodes represent the top 10 hub genes: The darker the color, the higher the score and the stronger the connection.

We uploaded the set of 118 overlapping target genes into the STRING database for protein-protein interaction (PPI) analysis. The results were utilized to construct a protein-protein interaction (PPI) network diagram using Cytoscape 3.10.1 software. This resulted in a network with 117 nodes (after excluding one unconnected node), 1265 edges, and an average node connectivity of 22.19, as shown in Fig 9. Subsequently, the CytoHubba plug-in was employed to identify and extract the top ten most significant genes based on their degree of connectivity within the network, as illustrated in Fig 10. These genes were AKT1, TNF, CASP3, EGFR, HSP90AA1, JUN, SRC, ESR1, PTGS2, and HSP90AB1. Topological parameters including node degree, betweenness, and closeness were summarized for each of these proteins in Table 2.

**Table 2:** Topological properties of the top ten hub genes with skin tissue expression score.

No.	Name	Target	Degree	Betweenness	Closeness
1	RAC-alpha serine/threonine-protein kinase	AKT1	74	0.08687	0.74342
2	Tumor necrosis factor	TNF	74	0.10399	0.73856
3	Caspase-3	CASP3	64	0.05704	0.68485
4	Epidermal growth factor	EGFR	61	0.04739	0.68072
5	Heat shock protein HSP 90-alpha	HSP90AA1	58	0.03466	0.64943
6	Transcription factor Jun	JUN	56	0.03096	0.65318
7	Proto-oncogene tyrosine-protein kinase	SRC	55	0.02987	0.64943
8	Heat shock protein HSP 90-beta	HSP90AB1	52	0.02565	0.62778
9	Prostaglandin G/H synthase 2	PTGS2	52	0.05570	0.64571
10	Estrogen receptor	ESR1	52	0.02611	0.64571

### 2.6. Docking study

**Fig 11:**
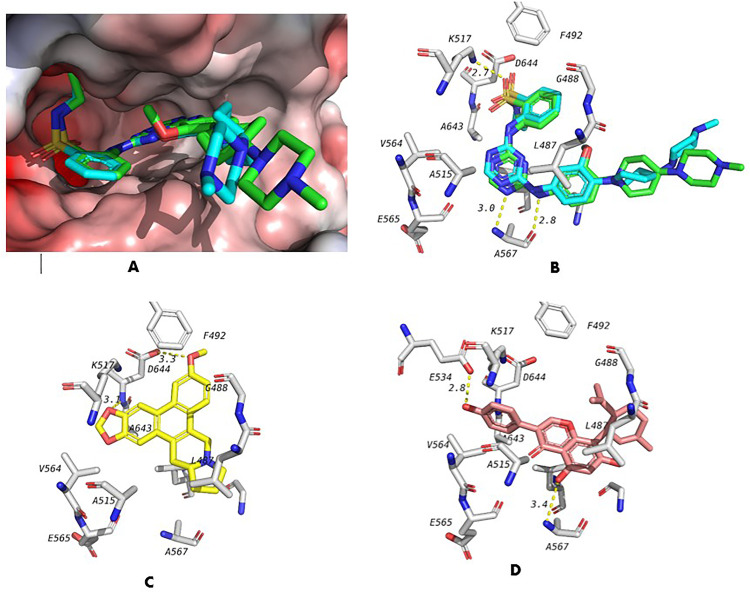
Results of docking study of identified compounds in the active site of fibroblast growth factor recptor (FGFR). **A)** Validation of docking procedure showing the docked co-crystalized ligand (Blue) overlapped with its crystal pose(green) Docking pose and interactions of the co-crystalized ligand. **C)** Docking pose and interactions of compound 19 (yellow). **D)** Docking pose and interactions of compound 17(pink).

Docking was done to investigate potential targets for compounds identified in the extract. The twenty annotated compounds were docked in the active sites of seven inflammatory mediators. This study was done to predict the potential mechanism for the effect of FCL extract when combined with indomethacin. The docking scores of these compounds against the seven targets are shown in Table 3. In addition to the test compounds, we also re-docked co-crystalized ligands to validate the used docking protocol and were accepted when RMSD between the crystal pose and docked pose are less than 2 Å. The docking score of the co-crystallized ligand was compared to the docking scores of test compounds. Several of the tested compounds showed scores better than the co-crystalized ligand in the case of fibroblast growth factor receptor (FGFR) and epidermal growth factor receptor (EGFR) targets. Furthermore, among tested compounds, compounds 2 and 17−20 showed best scores against these two targets. Against these targets, the best docking score was seen with the docking of Ficuseptin-C (19) in the active site of FGFR (−10.3 kcal/mol). Fig 11A shows the validation of the docking procedure through the re-docking of the co-crystalized ligand and it shows that the docking software was able to predict all the correct pose with good accuracy especially for the hydrogen bond part. The docking pose of the co-crystallized ligand Fig 11B showed the formation of 3 hydrogen bonds, two hydrogen bonds with A567 and one hydrogen bon with K517. Docking pose of compound 19 is shown in Fig 11C. The compound was docked in the same position of the co-crystalized ligand and was able to form 2 hydrogen bonds with residue sin the active site with D644. Ficusin-A (17) also showed a very good score (−10.2 kcal/mol) with the same target, FGFR. The docking pose of 17 in the active site of FGFR is shown in Fig 11D. The compound was also able to form 2 hydrogen bonds, a weak one with A567 which is similar to co-crystalized ligand and another one with E534. These findings suggest that the compounds may exert a potential effect on FGFR, which could contribute to their protective role against indomethacin-induced ulcers. Although further experimental studies are needed to confirm this hypothesis, these results pave the way for future investigative research.

**Table 3:** Docking scores of tested compounds.

		Docking Score (kcal/mol)						
		Anti-inflammatory targets						
		FGFR (6lvl)	COX2 (3ln1)	TNF-α (2az5)	TGF-βR (5e8s)	EGFR (1m17)	IGFR-1R (5fxs)	IL-1R1 (1g0y)
1	*F. latex*-peptide 3	−6.4	−7	−6.8	−7.4	−6.6	−6.4	−6.3
2	Ficine	−10	−9.7	−8.7	−9.1	−8.8	−9	−8.5
3	2-Acetyl-4-methylpyridine	−5.3	−6.4	−5.6	−5.8	−5	−5.3	−5.4
4	Aviprin-2-AC-3-ME	−8.3	−8.3	−7.8	−7.8	−7.6	−7.8	−7.1
5	Psoralenoside	−8.4	−8.3	−8	−8.8	−7.9	−7.8	−6.5
6	Caricaflavonol-diester-A	−6.6	−7.7	−6.9	−6.7	−6.1	−5.7	−6.1
7	Caricaflavonol-diester-B	−6.3	−7.7	−6.5	−7	−6.7	−6	−6.2
8	Aviprin	−7.9	−9.4	−7.2	−7.9	−7.3	−7.9	−7.2
9	Benganoic-acid	−7.8	−7.6	−7	−7.7	−7	−7	−7.2
10	Bergaptol	−7.2	−8	−6.6	−7.3	−6.9	−7	−6.6
11	Neobetanin	−8.6	−6.1	−8.2	−9	−8.9	−7.7	−8.8
12	Ficuformodiol-A	−8.3	−8.7	−7.4	−8.5	−8	−7.8	−7.5
13	Ficuisoflavone	−9	−8.5	−8.4	−9.9	−8.2	−8.4	−8.4
14	Ficuscarpanoside-B	−7.9	−7.5	−6.6	−8	−7.7	−7.1	−6.8
15	Ficuseptine	−8.9	−8.5	−7.8	−9	−8.3	−8.1	−7.9
16	Ficusic-acid	−6.1	−6.9	−6.2	−6	−6.1	−6.2	−5.7
17	Ficusin-A	−10.2	−7.5	−8.6	−8.9	−9.3	−7.8	−9.2
18	Ficusin-B	−9.6	−8.3	−8.5	−10	−8.7	−8	−9
19	Ficuseptin-C	−10.3	−11.3	−9.3	−10.2	−9.7	−10	−8.8
20	Ficuseptin-B	−10.1	−10.3	−9.4	−9.8	−9.5	−9.7	−8.6
	Co-crystalized Ligand	−9.2	−13.1	−9.1	---	−7.1	−10	---

## 3. Conclusion

Our primary focus in the present study is on determining if FCL extract has any gastroprotective effects. Twenty natural products were annotated as a result of metabolic profiling using HR- LC-MS. Further, male albino rats were used to test the FCLt’s gastro-protective efficacy in vivo. The ulcer index caused by indomethacin was greatly decreased due to the effects of FCL. Also, relative gene expression (IL-1, IL-6, and TNF-) was shown to be downregulated, which contributed to the reduction of inflammation generated by indomethacin. Furthermore, FCL significantly attenuated the increased relative gene expression of COX-2, TGF-, and IGF-1. In addition, FCL significantly increased EGF and KGF relative gene expression, showing its favorable effect on ulcer healing. In vitro tests involving hydrogen peroxide and superoxide radical scavenging established that FCL possessed significant antioxidant properties. Based on the top genes associated with peptic ulcers through pharmacological network analysis and conducting an in-silico assessment of the identified chemicals in FCL, such as Ficuseptin-C, Ficuseptin-B and Ficusin-A, could potentially account for the extract’s antiulcer properties. In this investigation, we saw that FCL has great promise as a natural gastro-protective agent.

## 4. Experimental

### 4.1. Plants material

Fresh FCL was obtained in May 2021 from the Sohag region of Egypt by meticulously extracting immature green fruits and the underside of a dark green leaf, drop-by-drop, without applying pressure, under identical meteorological and soil conditions. All latex samples used in this study were collected and preserved on ice throughout the collection period, which occurred in the early morning. At the conclusion of August, all samples were hand gathered at an equivalent stage of development. The latex fluid was transported to the laboratory under appropriate conditions and promptly kept at −20 °C until preserved at Deraya University. It was verified by Abdallah Salem (Minia, Egypt). A voucher specimen (LM 8–2021) is preserved in the Pharmacognosy Department, Faculty of Pharmacy, Deraya University, Egypt.

### 4.2. Sample preparation

The latex of *Ficus. Carica. L* was extracted with methanol and dried with a rotator evaporator at decreased pressure. The methanolic extract of *Ficus. Carica.L* latex (EFCL) was kept until investigation.

### 4.3. In vivo study

#### 4.3.1. Animal model.

This research used Wistar albino male rats with weights ranging from 150 to 200 grams. The study was approved by The Ethics Committee, Faculty of Pharmacy, Deraya University which stated that animals should not suffer at any stage of experimentation and be maintained in accordance with the Guide for the care and use of laboratory animals. The approval code is 20/2021. Comprising a 12-hour light/dark cycle, a controlled temperature of 24 °C, humidity levels of 60–65%, commercial pellet feed, and water. Prior to the commencement of the experiment, all animals were acclimated to the laboratory environment for a minimum duration of one week. The animals were accommodated in regular enclosures with unrestricted access to water and food. Rats were subjected to a 24-hour fast before the test to ensure gastric emptiness. Indomethacin (IND; 5 mg/kg, orally) was administered to induce gastric ulcers once daily over a duration of five days [42]. Rats were housed in cages with mesh bottoms to inhibit their food intake. With the exception of the last hour before the experiment, the animals had unrestricted access to water. To mitigate variations caused by diurnal cycles of possible regulators of gastric functions, all investigations were undertaken at a consistent time of day. The animals were randomly allocated into four groups, with six rats in each group [39].

For this antiulcer trial, the following groups were created:

Group A: Control (normal) – vehicle + vehicle (0.5 percent carboxymethyl cellulose (CMC) only)
Group B: (negative control) received 5 mg/kg IND + vehicle (0.5 percent CMC).
Group C: IND+Cimetidine
Group D: IND+ methanolic extract of *Ficus. Carica. L* latex.

An intraperitoneal injection of a ketamine-xylazine mixture was used to anesthetize the animals. (50 mg/kg body wt. ketamine and 10 mg/kg body wt. xylazine). All animals were euthanized by cervical dislocation after four hours. Following the excision of the stomachs and incisions along their greater curvature, the mucosal lesions were meticulously rinsed with tap water, normal saline was employed to eliminate all gastric contents, a macroscopic examination of the lesions was performed, the ulcer index (UI) was computed, and finally, histopathological preparation was executed [43,44].

#### 4.3.2. Evaluation of lesions in the gastric mucosa.

The ulcer index (U.I.) quantifies both the count and severity of lesions on the stomach’s mucosal lining. This index is determined through a scoring system ranging from 0 to 3, based on lesion size and severity. Lesions are categorized as follows: 0 indicates no lesions, 1 represents lesions smaller than 1 mm, 2 indicates lesions between 2–4 mm, and 3 represents lesions larger than 4 mm. Each rat’s lesion count is multiplied by the associated severity score to generate an individual lesion index. The U.I. for each group is then derived by averaging the lesion indices of all rats within that group [45]. To determine the drug’s protective effect, the preventative index (P.I.) is calculated following the formula established by Hano et al. [46].

### 4.4. Quantitative Real-Time Polymerase Chain Reaction (qRT-PCR)

Primer sequences of the genes included in the study (S2 Table), and the in vitro antioxidant activities (hydrogen peroxide scavenging activity and superoxide radical scavenging activity) are all described in detail in the supporting file

### 4.5. Histopathological examination

After determining the ulcer index, a histologic examination of the stomach was done. The corpus was removed and transferred to fresh formalin, which was then treated using standard procedures before being embedded in paraffin. Hematoxylin and eosin were used to stain 4-mm thick sections mounted on glass slides. An experienced pathologist who was blinded to the treatment checked coded slides.

### 4.6. Metabolic profiling

The analysis was conducted through liquid chromatography-mass spectrometry (LC-MS) on a Synapt G2 HDMS quadrupole time-of-flight hybrid mass spectrometer (Waters, Milford, USA). A 1 mg sample was loaded onto a BEH C18 column, kept at a steady temperature of 40°C, and secured with a guard column. A gradient elution was applied for the mobile phase, starting with 0.1% formic acid in water as the initial solvent (solvent A) and transitioning to pure acetonitrile (solvent B). For detailed differential analysis of the mass spectrometry data, MZmine 2.12 software was used, and the raw data were subsequently converted to mzML format files in both positive and negative ion modes using ProteoWizard. [47].

### 4.7. Network Pharmacology-based Analysis of *Ficus. Carica. L* latex extract in peptic ulcer

#### 4.7.1. Screening of *F.carica* latex extract related targets genes.

The target genes of compounds identified from *Ficus. Carica. L* latex extract were obtained by leveraging chemical similarities, pharmacophore models, and protein interactions through a search conducted on the BATMAN-TCM platform (http://bionet.ncpsb.org.cn/batman-tcm/) [48], and Swiss Target Prediction Database (http://www.swisstargetprediction.ch/). Subsequently, these target genes were converted to their canonical gene names using the UniProt database (https://www.uniprot.org/) [49].

#### 4.7.2. Screening of peptic ulcer related genes.

To identify genes associated with peptic ulcer disease, data were collected from several databases, including GeneCards (https://www.genecards.org/) [25], the Comparative Toxicogenomics Database (CTD) (https://ctdbase.org/), and DisGeNET (https://www.disgenet.org/), using “peptic ulcer” as the search term and restricting the species to Homo sapiens. After removing duplicates, a comparison was conducted to detect overlapping proteins linked to both the disease and related compounds, using InteractiVenn (http://www.interactivenn.net/) [26]. This F facilitated the identification of proteins potentially involved in the wound healing processes targeted by these compounds.

#### 4.7.3. Protein–Protein Interaction (PPI) Network Construction.

A protein-protein interaction (PPI) network was developed using STRING version 12.0 (https://string-db.org/) [27], incorporating a selected list of target genes to examine potential molecular interactions. This network was subsequently imported into Cytoscape software version 3.10.1 (USA) [28], a robust, open-access tool designed for visualizing, modeling, and conducting in-depth analysis of molecular and genetic networks. The network was generated with a confidence score threshold of 0.400 to ensure meaningful interactions. To pinpoint the most crucial genes within the network, the CytoHubba plug-in in Cytoscape was employed, enabling the identification of the top 10 key genes with significant roles in the interaction framework.

### 4.8. Docking study

The three-dimensional (3D) structures of the selected compounds were acquired from PubChem (https://pubchem.ncbi.nlm.nih.gov/) and subsequently optimized and converted to the pdbqt format using the PyRx software suite, facilitating compatibility with docking tools. Target protein structures were retrieved from the Protein Data Bank (https://www.rcsb.org/) and prepared for docking in PyRx using the macromolecule conversion script. The protein targets included key receptors and enzymes: fibroblast growth factor receptor (FGFR, PDB ID: 6lvl), cyclooxygenase-2 (COX-2, PDB ID: 3ln1), tumor necrosis factor alpha (TNF-alpha, PDB ID: 2az5), transforming growth factor β receptor (TGF-βR, PDB ID: 5e8s), epidermal growth factor receptor (EGFR, PDB ID: 1m17), insulin-like growth factor receptor (IGFR, PDB ID: 5fxs), and interleukin-1 receptor (IL-1R, PDB ID: 1g0y). Docking simulations were carried out using AutoDock Vina [29] within a 25 Å cubic grid box centered on the co-crystallized ligand, with the exhaustiveness parameter set to 16 for thorough exploration, as previously outlined [30]. To ensure accuracy, the docking protocol was validated by redocking the native ligand into the active site of each target protein, accepting results with a root-mean-square deviation (RMSD) below 2 Å, in line with prior validation criteria [31].

### 4.9. Statistical analysis

Data were presented as the mean with standard deviation (Mean ± SD) derived from six independent samples (n = 6). To evaluate statistical differences, a one-way analysis of variance (ANOVA) was conducted, followed by Dunnett’s multiple comparison test, alongside a two-way ANOVA to account for interactions between variables. All statistical computations were performed using GraphPad Prism version 7 (GraphPad Software, San Diego, CA, USA). A p-value of less than 0.05 (p < 0.05) was considered statistically significant, with significant results indicated by the symbols * or #.

## Supporting information

S1 File Supplemental Tables, and captions. (DOCX)
