# Supplementary material for: Network pharmacology reveals Ficus. Carica. L latex as a potential therapeutic agent for gastric ulcers by modulating inflammation and promoting repair
Source: PLoS One. 2025 Dec 2;20(12):e0333777. doi: 10.1371/journal.pone.0333777 (PMC12671817; doi:10.1371/journal.pone.0333777)
Supplement: S1 File — (DOCX) [file pone.0333777.s001.docx]

**Table S1:** **Dereplicated identified compounds of *Ficus Carica.l Latex* extract.**

| **Ref** | **Biological source** | **Molecular Formula** | **Rt** | **Exact mass** | **Name** | **No** |
| --- | --- | --- | --- | --- | --- | --- |
| [1] | Latex  *Ficus carica* | C_17_H_34_N_6_O_4_ | 5.25 | 386.264154 | Ficus Latex peptide **3** | 1  11 |
| [2] | Latex  *Ficus carica* | C_20_H_19_NO_4_ | 3.99 | 337.38 | Ficine | 2 |
| [3] | *Ficus carica* | C_8_H_9_NO | 2.390 | 135.068 | 2-Acetyl-4-methylpyridine | 3 |
| [4] | *Ficus carica* | C_19_H_20_O_7_ | 3.85 | 360.120905 | Aviprin  E- form 3'-Me ether, 2'-Ac | 4 |
| [5] | *Ficus carica* | C_17_H_18_O_9_ | 3.75 | 366.095085 | Psoralenoside. | 5 |
| [6] | *Ficus carica* | C_51_H_76_O_8_ | 3.2 | 816.55402 | Caricaflavonol diester A. | 6 |
| [6] | *Ficus carica* | C_51_H_76_O_9_ | 3.22 | 832.548935 | Caricaflavonol diester B. | 7 |
| [7] | *Ficus pumila* | C_16_H_16_O_6_ | 5.08 | 304.0946 | Aviprin | 8 |
| [8] | *Ficus bengalensis* | C_18_H_26_O_5_ | 2.62 | 322.178025 | Benganoic acid | 9 |
| [9] | *Ficus Religiosa* | C_11_H_6_O_4_ | 1.76 | 202.02661 | Bergaptol | 10 |
| [10] | *Ficus indica* | C_24_H_24_N_2_O_13_ | 3.76 | 548.127843 | Neobetanin. | 11 |
| [11] | *Ficus formosana* | C_15_H_14_O_5_ | 2.37 | 274.084125 | Ficuformodiol A | 12 |
| [12] | *Ficus microcarpa* | C_20_H_18_O_6_ | 2.19 | 354.11034 | Ficuisoflavone | 13 |
| [13] | *Ficus microcarpa* | C_17_H_24_O_10_ | 5.25 | 388.13695 | Ficuscarpanoside B. or  Cremanthodioside | 14 |
| [14] | *Ficus septica* | C_22_H_22_NO_2_ 1+ | 4.59 | 332.165054 | Ficuseptine | 15 |
| [15] | *Ficus microcarpa* | C_11_H_16_O_3_ | 2.15 | 196.109945 | Ficusic acid | 16 |
| [16] | *Ficus septica* | C_25_H_24_O_5_ | 2.310 | 404.1623 | Ficusin A | 17 |
| [16] | *Ficus septica* | C_25_H_24_O_6_ | 2.59 | 420.1572 | Ficusin B | 18 |
| [17] | *Ficus septica* | C_22_H_21_NO_3_ | 4.04 | 347.1521 | Ficuseptine C | 19 |
| [17] | *Ficus septica* | C_23_H_23_NO_4_ | 1.34 | 377.1627 | Ficuseptine B. | 20 |

**Quantitative Real-Time Polymerase Chain Reaction (qRT-PCR)**

Approximately 50 mg of the gastric mucosa tissue was homogenized by ultrasonic homogenizer (SFX 550 Branson Digital Sonifier® ultrasonic cell disruptor/homogenizer is versatile. Danbury, CT, USA) in 0.5 mL of TRIzol TM reagent (Amresco, Solon, OH, USA). RNA was extracted from gastric mucosa tissue using the TRIzol TM RNA Extraction Reagent (Amresco, Solon, OH, USA) as instructed by the manufacturer. The overall RNA concentration was estimated at A260 nm, and the purity was measured based on the ratio A260/A280. Samples with purity ≥ 1.7 were used for qRT-PCR. GAPDH was used as a reference housekeeping gene. cDNA synthesis was performed for equivalent amounts of total RNA in all samples using the RevertAid H Minus First Strand cDNA Synthesis Kit (#K1632, Thermo Science Fermentas, St. Leon-Ro, Germany) as directed by the manufacturer. Real-time PCR was conducted with single-stranded cDNAs. The sequences of the used primers are shown in Table 1 PCR reactions were conducted by SYBER Green (#K0251, Thermo Scientific Fermentas St. Leon-Ro, Germany-Maxima SYBER Green qPCR Master Mix (2X)) using a StepOne Real-Time PCR Detection System (Applied Biosystems).

**Table S2:** **Primer sequence of genes included in the study**

| Primer | Sequence 5' to 3' |
| --- | --- |
| *TNF-α* | Forward CAG AGG GAA GAG TTC CCC AG  Reverse CCT TGG TCT GGT AGG AGA CG |
| *IL-1β* | Forward GAG GCT GAC AGA CCC CAA AAG AT  Reverse GCA CGA GGC ATT TTT GTT GTT CA |
| *IL-6* | Forward CGC CCT AGT GGG GTA GTA GT  Reverse CAG TCA GAC CCT TCA CCG TC |
| *TGF-β* | Forward TTG CCC TCT ACA ACC AAC ACA A  Reverse GCT TGC GAC CCA CGT AGT A |
| *COX-2* | Forward CAT TGA CCA GAG CAG AGA GAT  Reverse TTC TTG AAT GTC CTC TCT TTC |
| *IGF-1* | Forward CCT CAT TAT CCC TGC CCA CCA A  Reverse GCT GGT GAA GGT GAG CAA GC |
| *KGF* | Forward CCC AGG AGA TGA GGA ACA GC  Reverse TCT TCG TTT TCT TCC CTT TGA CAG |
| *EGF* | Forward CGC CGC AGA CTT ACC CAG AA  Reverse ACC TGG TTT TGC CAA TGG GTG |
| *GAPDH* | Forward ACC AAC TGC TTA GCC CCC C  Reverse GCA TGT CAG ATC CAC AAC GG |

Real-time polymerase chain reaction (qRT-PCR) achieved using 20 µl of RealMOD Green qRT-PCR Mix kit (iNtRON biotechnology) with 0.02 µg RNA per reaction and 10 Pmol of unique primers, for 30 cycles of 95 ^◦^C for 10 s. and 60 ^◦^C for 1 min. The comparative Ct (threshold cycle) approach was used to assess the relative concentrations of the products. The relative expression was determined using formula 2(−^∆∆Ct^)[18]. They were scaled compared to the controls.

**In Vitro Antioxidant Activity**

**Hydrogen Peroxide Scavenging Activity**

The reaction with a defined amount of exogenously provided H_2_O_2_ was used to determine the hydrogen peroxide (H_2_O_2_) scavenging activity that reflects the anti-oxidative capacity of *Ficus Carica latex extract*. Colorimetric analysis was used to estimate the residual H_2_O_2_ [19]. In brief, 20µl of the extract mixed with 500 µl of H_2_O_2_ and incubated at 37ºC for 10 minutes. After that, 500 µl of enzyme/3, 5-dichloro-2-hydroxyl-benzenesulfonate solution added and incubated at 37ºC for 5 minutes. Colorimetrically, the intensity of the colored product measured at 510 nm. Positive control was ascorbic acid. The percentage of H_2_O_2_ scavenging activity was determined by comparing the results of the test with those of the control using the following formula:

$$scavenging activity=\frac{A control -A sample}{A control}\times100$$

IC_50_ of each sample calculated after performing the assay at four different concentrations (1000 µg/mL, 500 µg/mL, 250 µg/mL, and 125 µg/mL) using Graph pad prism 7 software.

**Superoxide Radical Scavenging Activity**

The superoxide anion scavenging activity was measured as described by Sreenivasan et al.2007 [20]. The superoxide anion radicals were formed in a Tris – HCl buffer (16 mM, pH8.0) containing 90 µl of NBT (0.3 mM), 90 µL of NADH (0.936 mM), 0.1mL of *Ficus Carica latex extract* (125, 250, 500, and 1000 g/mL), and 0.8 mL Tris – HCl buffer (16 mM, PH 8.0). The reaction initiated by adding 0.1 mL Phenazine Methosulfate (PMS) solution (0.12 mM) to the mixture, which was then incubated at 25˚C for 5 minutes, and at 560 nm, the absorbance was measured. Ascorbic acid was selected as a reference. The percentage inhibition was obtained by comparing the test findings to those of the control using the formula below:

$$Superoxide scavenging activity=\frac{A control -A sample}{A control}\times100$$

IC_50_ was calculated using Graph pad prism 7 software by performing the test at four different concentrations.

**References**

1. Maruyama, S., S. Miyoshi, and H. Tanaka, *Angiotensin I-Converting Enzyme Inhibitors Derived from Ficus carica.* Agricultural and Biological Chemistry, 1989. **53**(10): p. 2763-2767.

2. Johns, S.R., J.H. Russel, and M.L. Heffernan, *Ficine, A novel flavonoidal alkaloid from Ficus pantoniana.* Tetrahedron Letters, 1965. **6**(24): p. 1987-1991.

3. Kaiser, R., *New natural products of structural and olfactory interest identified in fig leaf absolute (Ficus carica L.)*, in *Progress in Essential Oil Research: Proceedings of the International Symposium on Essential Oils, Holzminden/Neuhaus, Federal Republic of Germany, Sept. 18–21, 1985*, B. Ernst-Joachim, Editor. 2019, De Gruyter. p. 227-240.

4. Al-Musayeib, N., et al., *Chemotaxonomic Diversity of Three Ficus Species: Their Discrimination Using Chemometric Analysis and Their Role in Combating Oxidative Stress.* Pharmacognosy magazine, 2017. **13**(Suppl 3): p. S613-S622.

5. Alqasoumi, S., et al., *Phytochemical and Pharmacological study of Ficus palmata growing in Saudi Arabia.* Saudi Pharmaceutical Journal, 2013. **27**.

6. Ahmad, M.Z., M. Ali, and S.R.J.A.J.B.P.S. Mir, *Anti-diabetic activity of Ficus carica L. stem barks and isolation of two new flavonol esters from the plant by using spectroscopical techniques.* 2013. **3**(18): p. 22-8.

7. Al-Musayeib, N., et al., *Chemotaxonomic diversity of three Ficus species: their discrimination using chemometric analysis and their role in combating oxidative stress.* 2017. **13**(Suppl 3): p. S613.

8. Sultana, S., et al., *Chemical Constituents From the Aerial Roots of Ficus benghalensis L., Leaves of Nyctanthes arbor-tristis L. and Roots of Verbesina encelioides (Cav.) Benth. et Hook. f.* 2018: p. 16-26.

9. Jangde, R.K.J.R.J.o.S. and Technology, *Plant Profile of Ficus religosa: A Review.* 2015. **7**(4): p. 193.

10. Strack, D., U. Engel, and V.J.P. Wray, *Neobetanin: a new natural plant constituent.* 1987. **26**(8): p. 2399-2400.

11. Sheu, Y.-W., et al., *Cytotoxic flavonoids and new chromenes from Ficus formosana f. formosana.* 2005. **71**(12): p. 1165-1167.

12. Li, Y.-C. and Y.-H.J.J.o.N.P. Kuo, *Two new isoflavones from the bark of Ficus microcarpa.* 1997. **60**(3): p. 292-293.

13. Ouyang, M.-A. and Y.-H.J.J.o.A.n.p.r. Kuo, *Water-soluble constituents from aerial roots of Ficus microcarpa.* 2006. **8**(7): p. 625-630.

14. Wu, P.-L., et al., *Phenanthroindolizidine alkaloids and their cytotoxicity from the leaves of Ficus septica.* 2002. **57**(12): p. 2401-2408.

15. Li, Y.-c. and Y.-H.J.P. Kuo, *A monoterpenoid and two simple phenols from heartwood of Ficus microcarpa.* 1998. **49**(8): p. 2417-2419.

16. Aida, M., Y. Hano, and T.J.H. Nomura, *FICUSINS A AND B, TWO NEW CYCLIC-MONOTERPENE-SUBSTITUTED ISOKAVONES FROM FICUSSEPTICA BARM. F.'.* 1995. **41**(12): p. 2761.

17. Damu, A.G., et al., *Phenanthroindolizidine alkaloids from the stems of Ficus septica.* 2005. **68**(7): p. 1071-1075.

18. VanGuilder, H.D., K.E. Vrana, and W.M. Freeman, *Twenty-five years of quantitative PCR for gene expression analysis.* Biotechniques, 2008. **44**(5): p. 619-626.

19. Hassan, H. and A. Abdel-Aziz, *Evaluation of free radical-scavenging and anti-oxidant properties of black berry against fluoride toxicity in rats.* Food and chemical toxicology, 2010. **48**(8-9): p. 1999-2004.

20. Srinivasan, R., et al., *Antioxidant activity of Caesalpinia digyna root.* Journal of Ethnopharmacology, 2007. **113**(2): p. 284-291.
